# Supplementary material for: DNA requirement in FANCD2 deubiquitination by USP1-UAF1-RAD51AP1 in the Fanconi anemia DNA damage response
Source: Nat Commun. 2019 Jun 28;10:2849. doi: 10.1038/s41467-019-10408-5 (PMC6599204; doi:10.1038/s41467-019-10408-5)
Supplement: Supplementary file 1 — Supplementary Information [file 41467_2019_10408_MOESM1_ESM.pdf]

Supplementary Information

DNA Requirement in FANCD2 Deubiquitination by USP1-UAF1-RAD51AP1 in the Fanconi anemia DNA Damage Response

Liang, *et al*

Supplementary Table 1. Primers used in this study

Primer Name	Sequence (listed 5'-3')
UAF1^{R30A}-FWD	CAG AGC ATT GAC TCC ATT TGC GTT GTA CTT CTC CAC TTC A
UAF1^{R30A}-REV	TGA AGT GGA GAA GTA CAA CGC AAA TGG AGT CAA TGC TCT G
UAF1^{R50A}-FWD	CTA AAT AGA CTT TTC ACA GCC GGT GCA GAC TCT ATC ATA AGA ATA TGG
UAF1^{R50A}-REV	CCA TAT TCT TAT GAT AGA GTC TGC ACC GGC TGT GAA AAG TCT ATT TAG
UAF1^{K168A}-FWD	TGG CCA GGC TAT AAA TGG AAT CTG CGT TTC CAC TTA AAG AAG AAG TTG
UAF1^{K168A}-REV	CAA CTT CTT CTT TAA GTG GAA ACG CAG ATT CCA TTT ATA GCC TGG CCA

Supplementary Table 2. Oligonucleotides used in this study

Name	Sequence (listed 5'-3')	Assay
P1	TTATATCCTTTACTTTGAATTCTATGTTTAACCTTTTACTTAT TTTGTATTAGCCGGATCCTTATTTCAATTATGTTCAT	DNA binding
P2	ATGAACATAATTGAAATAAGGATCCGGCTAATACAAAATAA GTAAAAGGTTAAACATAGAATTCAAAGTAAAGGATATAA	DNA binding
D1	CATTGCATATTTAAAACATGTTGGAAGGCTCGATGCATGC TGATAGCCTACTAGTGCTGCTGGCTTTCAAATGACCTCTT ATCAAGTGAC	DNA binding
D2	GTCACTTGATAAGAGGTCATTTGAATTCATGGCTTAGAGCT TAATTGCTGAATCTGGTGCTGGGATCCAACATGTTTTAAAT ATGCAATG	DNA binding
D3	CTGCTACGATGCTAGTCGTAGCTCGGCAGTCGTAGCAGGT TCCCAGCACCAGATTCAGCAATTAAGCTCTAAGCCATGAA	DNA binding
64-mer	TTTCCCAGCACCAGATTCAGCATACGTTACCGATCGTACG TTCGATGCTGGCTACTGCTAGCTT	Ubiquitination

Supplementary Table 3. Summary of UAF1 and RAD51AP1 mutants

Mutants	Activity	Reference
$UAF1^{11A}$ (Residues R30, R50, K117, T161, K168, S230, R272, R274, K275, K318 and I363 converted to A)	DNA Binding Deficient	This Study
$UAF1^{3A}$ (Resides R30, R50 and K168 converted to A)	DNA Binding Deficient	This Study
$UAF1^{436X}$	DNA Binding Proficient	Liang et al., 2016
$UAF1^{SLD}$	DNA Binding Deficient	Liang et al., 2016
$RAD51AP1^{DM}$ (N-K6RA/C-K7WA)	DNA Binding Deficient	Dunlop et al., 2012

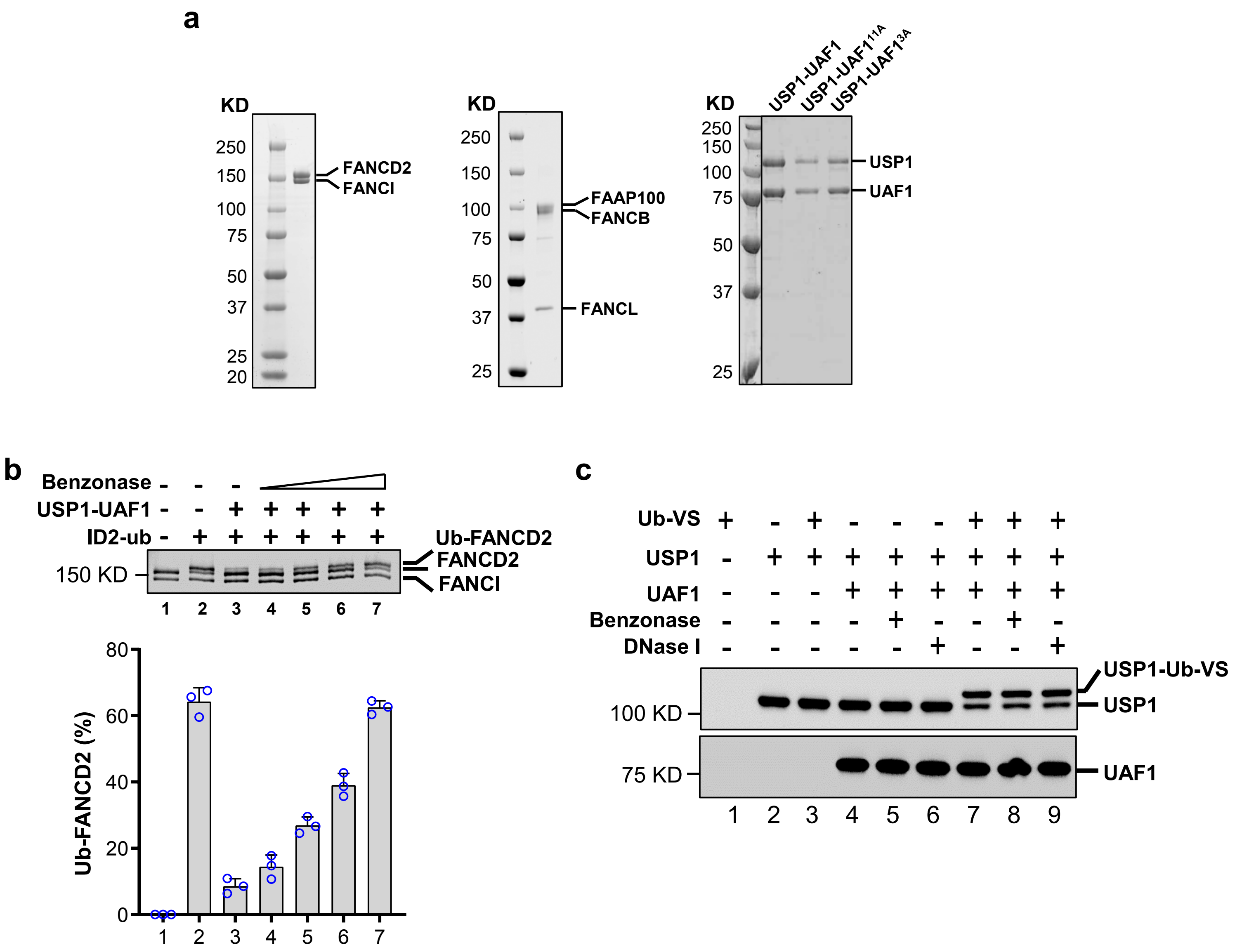

Supplementary Figure 1 Purified proteins used for FANCD2 ubiquitination and deubiquitination.
a, Purified ID2, BL100 and wild type or mutant forms USP1-UAF1 were analyzed by SDS-PAGE with Coomassie Blue staining.
b, Effect of benzonase (0.01, 0.04, 0.08, 0.2 U; lanes 4-7) on FANCD2 deubiquitination. The error bars represent mean values + S.D. of data from three independent experiments.
c, The DUB activity of purified USP1-UAF1 was monitored using the Ub-VS probe without or with benzonase or DNase I. UAF1 and USP1 were visualized by Western blotting.

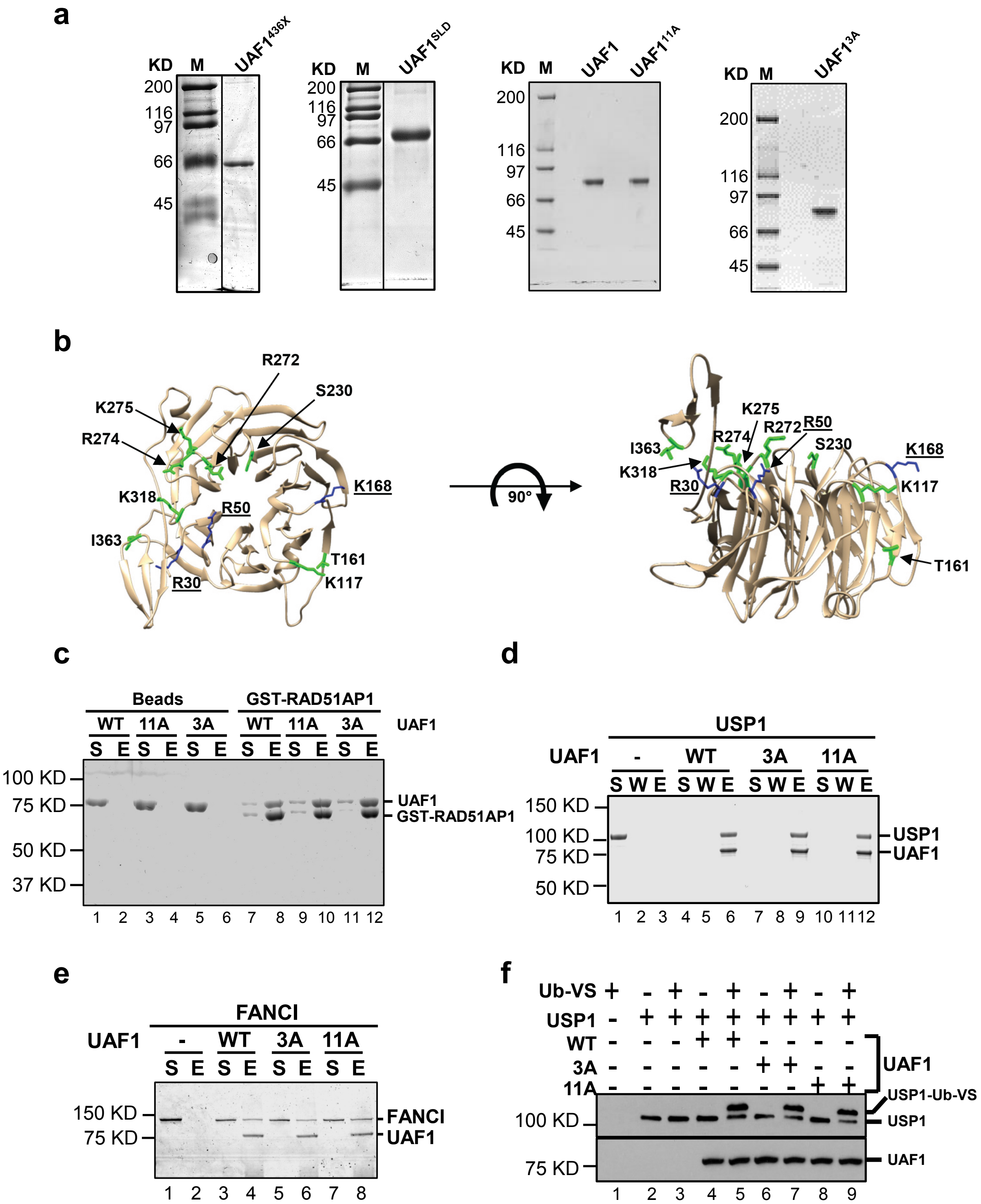
a
KD
M
UAF1436X
200
116
97
66
45
UAF1SLD
UAF1
UAF111A
UAF13A
b
R272
S230
K275
R274
K318
R50
K168
I363
R30
T161
K117
90°
c
Beads
GST-RAD51AP1
WT
11A
3A
UAF1
S
E
100 KD
75 KD
50 KD
37 KD
GST-RAD51AP1
1 2 3 4 5 6 7 8 9 10 11 12
d
USP1
-
W
150 KD
USP1
e
FANCI
f
Ub-VS
USP1-Ub-VS
100 KD
75 KD
1 2 3 4 5 6 7 8 9

Supplementary Figure 2 Construction and characterization of UAF1 mutants

a, Purified UAF1, UAF1^{436X}, UAF1^{SLD}, UAF1^{11A} and UAF1^{3A} were analyzed by SDS-PAGE with Coomassie Blue staining.

b, Cartoon representation of the crystal structure of the UAF1 WD40 repeat domain highlighting the amino acid residues changed in the 3A (underlined) and 11A (underlined and not underlined residues) mutants.

c, GST-tagged RAD51AP1 was tested for interaction with wild type and mutant UAF1 proteins. Glutathione resin was used in the pulldown. S, supernatant containing unbound proteins; E, SDS eluate of the affinity resin.

d, Strep II-tagged wild type and mutant UAF1 proteins were tested for USP1 interaction. Strep-Tactin resin was used in the pulldown. S, supernatant containing unbound proteins; W, buffer wash of the affinity matrix; E, SDS eluate of the affinity resin.

e, Strep II-tagged UAF1 and mutants were tested for FANCI interaction. Strep-Tactin resin was used in the pulldown. S, supernatant containing unbound proteins; E, SDS eluate of the affinity resin.

f, Stimulation of USP1 DUB activity by UAF1 DNA binding mutants. The DUB activity of purified USP1 and UAF1 (WT), UAF1^{3A} (3A) and UAF1^{11A} (11A) was monitored using the Ub-VS probe as in Supplemental Figure 1c.

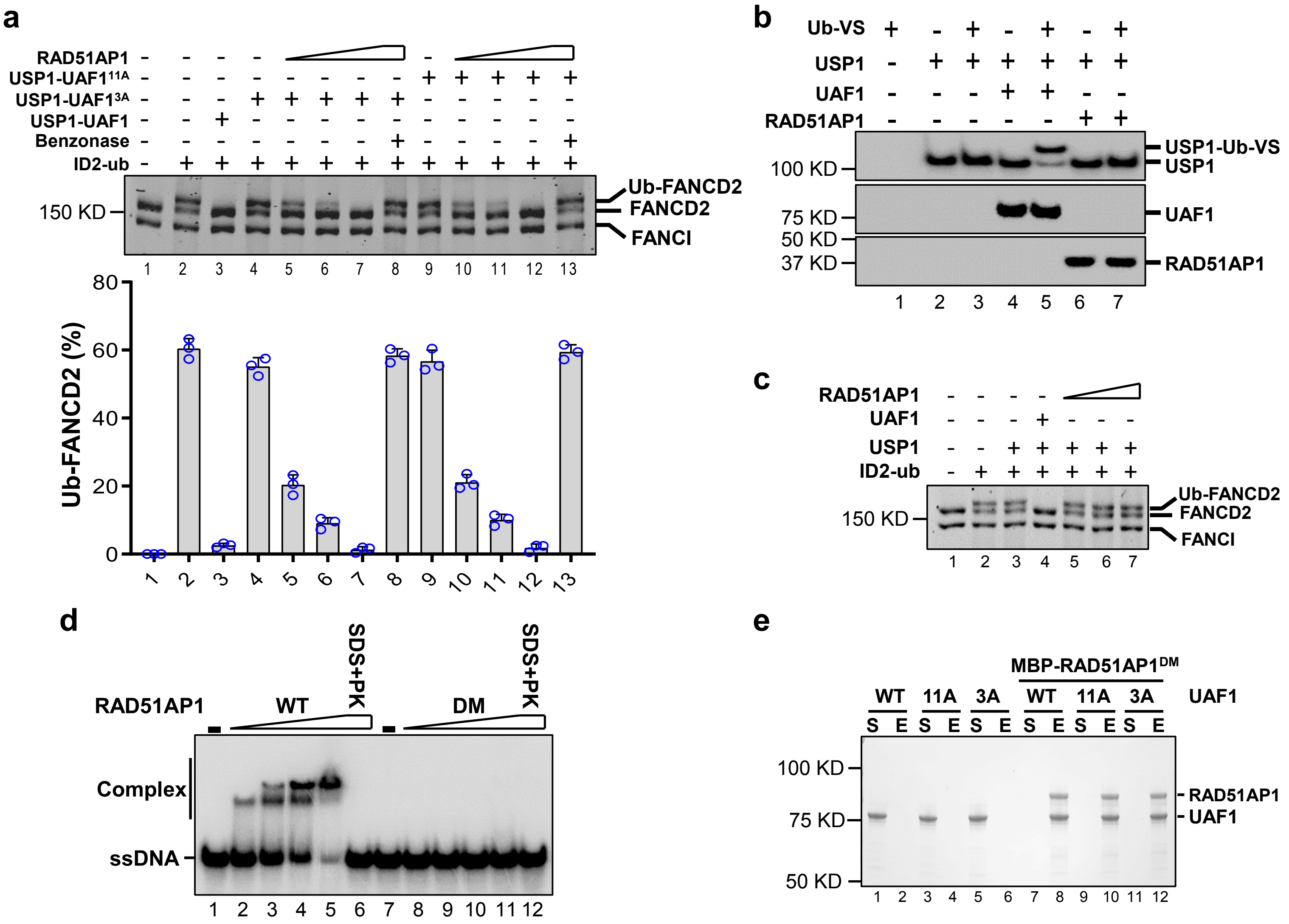

Supplementary Figure 3 DNA targeting of USP1-UAF1 by RAD51AP1 in FANCD2 deubiquitination.
a, RAD51AP1 was tested in FANCD2 deubiquitination reactions with USP1-UAF1, USP1-UAF1^{3A} and USP1-UAF1^{11A}. The error bars represent the mean + S.D. of data from three independent experiments.
b, In the absence of UAF1, RAD51AP1 does not stimulate USP1 activity. The DUB activity of purified USP1 and RAD51AP1 or UAF1 was monitored using the Ub-VS probe as in Supplemental Figure 1c. UAF1, USP1, and RAD51AP1 were visualized by Western blotting.
c, RAD51AP1 was tested in FANCD2 deubiquitination reactions with USP1.
d, RAD51AP1 and RAD51AP1^{DM} (10, 25, 50 and 100 nM) were examined for ssDNA binding. SDS+PK: SDS and proteinase K treatment to digest protein.
e, MBP-tagged RAD51AP1^{DM} was tested for interaction with UAF1, UAF1^{3A} and UAF1^{11A}. Protein complexes were captured on amylose resin. S, supernatant containing unbound proteins; E, SDS eluate of the affinity resin.

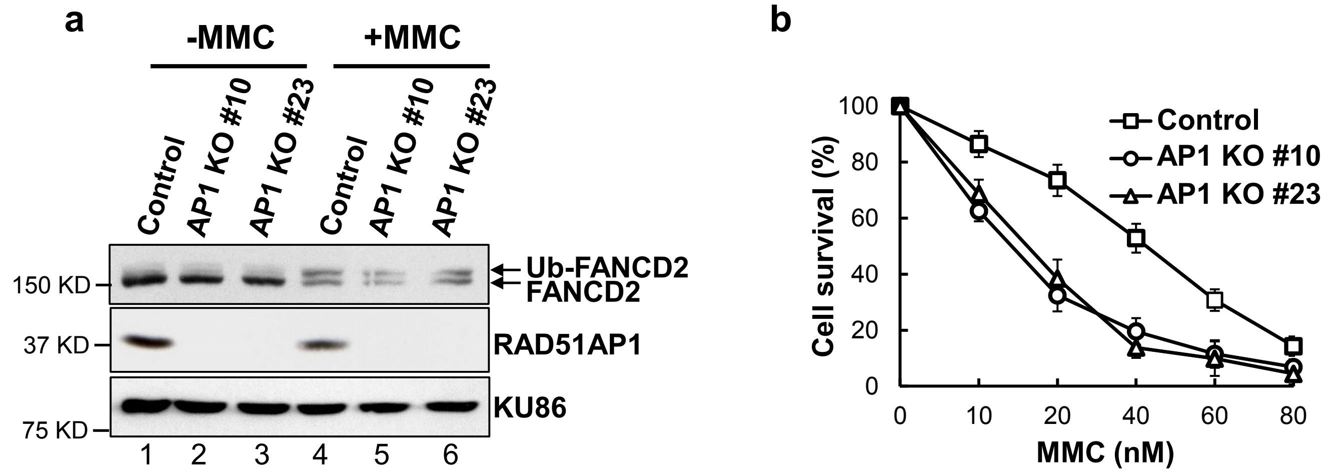

Supplementary Figure 4 Examination of RAD51AP1 null cells for FANCD2 ubiquitination and MMC sensitivity.

a, FANCD2 ubiquitination was examined in RAD51AP1 knockout cell lines (#10 and # 23) with or without MMC treatment (1 μM for 16 h).

b, Survival of RAD51AP1 knockout cells (clone #10 or #23) after MMC treatment. The error bars represent the mean ± S.D. of data from three independent experiments.
