# Supplementary material for: DNA requirement in FANCD2 deubiquitination by USP1-UAF1-RAD51AP1 in the Fanconi anemia DNA damage response
Source: Nat Commun. 2019 Jun 28;10:2849. doi: 10.1038/s41467-019-10408-5 (PMC6599204; doi:10.1038/s41467-019-10408-5)
Supplement: Supplementary file 3 — Reporting Summary [file 41467_2019_10408_MOESM3_ESM.pdf]

nature research

Corresponding author(s): Patrick Sung, Gary Kupfer

Last updated by author(s): Apr 18, 2019

Reporting Summary

Nature Research wishes to improve the reproducibility of the work that we publish. This form provides structure for consistency and transparency in reporting. For further information on Nature Research policies, see Authors & Referees and the Editorial Policy Checklist.

Statistics

For all statistical analyses, confirm that the following items are present in the figure legend, table legend, main text, or Methods section.

n/a	Confirmed	
☐	☒	The exact sample size (n) for each experimental group/condition, given as a discrete number and unit of measurement
☐	☒	A statement on whether measurements were taken from distinct samples or whether the same sample was measured repeatedly
☐	☒	The statistical test(s) used AND whether they are one- or two-sided<br>*Only common tests should be described solely by name; describe more complex techniques in the Methods section.*
☐	☒	A description of all covariates tested
☒	☐	A description of any assumptions or corrections, such as tests of normality and adjustment for multiple comparisons
☐	☒	A full description of the statistical parameters including central tendency (e.g. means) or other basic estimates (e.g. regression coefficient) AND variation (e.g. standard deviation) or associated estimates of uncertainty (e.g. confidence intervals)
☒	☐	For null hypothesis testing, the test statistic (e.g. F, t, r) with confidence intervals, effect sizes, degrees of freedom and P value noted<br>*Give P values as exact values whenever suitable.*
☒	☐	For Bayesian analysis, information on the choice of priors and Markov chain Monte Carlo settings
☒	☐	For hierarchical and complex designs, identification of the appropriate level for tests and full reporting of outcomes
☒	☐	Estimates of effect sizes (e.g. Cohen's d, Pearson's r), indicating how they were calculated

Our web collection on statistics for biologists contains articles on many of the points above.

Software and code

Policy information about availability of computer code

Data collection	UAF1 protein sequence is from NCBI (Reference No. NP_065890.1); UAF1 and DDB2 structures are from Protein Data Bank (PDB accession code: 3EI2 and 5CVL).
Data analysis	Online analysis software CCP4 (http://www.ccp4.ac.uk); ConSeq neural-network algorithm (http://consurf.tau.ac.il/); Chimera (1.10.2); Quantity One (4.6.2)

For manuscripts utilizing custom algorithms or software that are central to the research but not yet described in published literature, software must be made available to editors/reviewers. We strongly encourage code deposition in a community repository (e.g. GitHub). See the Nature Research guidelines for submitting code & software for further information.

Data

Policy information about availability of data

All manuscripts must include a data availability statement. This statement should provide the following information, where applicable:

- Accession codes, unique identifiers, or web links for publicly available datasets
- A list of figures that have associated raw data
- A description of any restrictions on data availability

All related data of the study are readily available from us upon request.

Field-specific reporting

Please select the one below that is the best fit for your research. If you are not sure, read the appropriate sections before making your selection.

☒ Life sciences ☐ Behavioural & social sciences ☐ Ecological, evolutionary & environmental sciences

For a reference copy of the document with all sections, see nature.com/documents/nr-reporting-summary-flat.pdf

Life sciences study design

All studies must disclose on these points even when the disclosure is negative.

Sample size	No sample size calculation was performed. We used the data from at least 3 independent experiments. Three repeats is usually sufficient for evaluating the spread of the data in our assay.
Data exclusions	No data were excluded for analysis.
Replication	The experimental findings were reliably reproduced and the data are analyzed from at least three independent experiments.
Randomization	No randomization was used for our biochemistry and cell-based assay.
Blinding	Group allocation and outcome assessment were done in a fully blinded manner.

Reporting for specific materials, systems and methods

We require information from authors about some types of materials, experimental systems and methods used in many studies. Here, indicate whether each material, system or method listed is relevant to your study. If you are not sure if a list item applies to your research, read the appropriate section before selecting a response.

Materials & experimental systems

n/a	Involved in the study
☐	☒ Antibodies
☐	☒ Eukaryotic cell lines
☒	☐ Palaeontology
☒	☐ Animals and other organisms
☒	☐ Human research participants
☒	☐ Clinical data

Methods

n/a	Involved in the study
☒	☐ ChIP-seq
☒	☐ Flow cytometry
☒	☐ MRI-based neuroimaging

Antibodies

Antibodies used	Anti-RAD51AP1 antibody: Mouse polyclonal to RAD51AP1, abcam (ab88370);<br>Anti-USP1 antibody: Rabbit polyclonal to USP1, abcam (ab108104);<br>Anti-UAF1 antibody: Rabbit polyclonal to WDR48, Thermo Fisher (cat no: PA5-24007);<br>Anti-FANCD2 antibody: Mouse Monoclonal (Clone FI17), Santa Cruz Biotechnology (sc-20022);<br>Anti-KU86 antibody: Mouse Monoclonal (Clone B-4), Santa Cruz Biotechnology (sc-515736)
Validation	The primary antibodies were validated for use based on the position of the antigen in the SDS-PAGE gels and the disappearance upon shRNA knock down in our assay.

Eukaryotic cell lines

Policy information about cell lines

Cell line source(s)	HeLa cell line was purchased from ATCC and stored in our lab.
Authentication	The cell lines used have not been authenticated by us.
Mycoplasma contamination	All the cell lines were tested negative for mycoplasma contamination.
Commonly misidentified lines<br>(See ICLAC register)	No commonly misidentified cell lines were used.
