# Supplementary material for: DNA requirement in FANCD2 deubiquitination by USP1-UAF1-RAD51AP1 in the Fanconi anemia DNA damage response
Source: Nat Commun. 2019 Jun 28;10:2849. doi: 10.1038/s41467-019-10408-5 (PMC6599204; doi:10.1038/s41467-019-10408-5)

Figure 1b

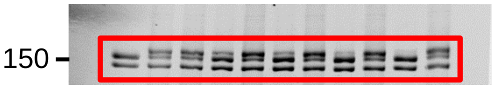

Figure 1c

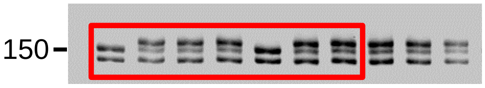

Figure 2b

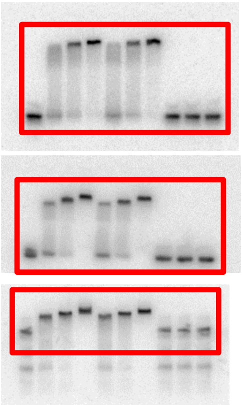

Figure 2c

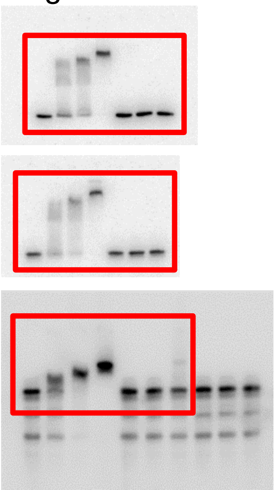

Figure 2d

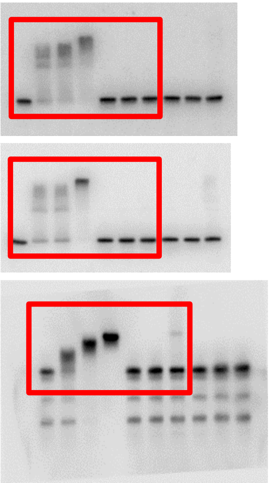

Figure 3a

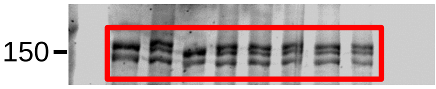

Figure 3b

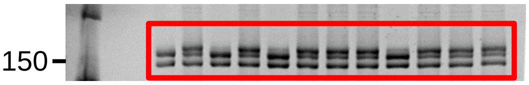

Figure 4a

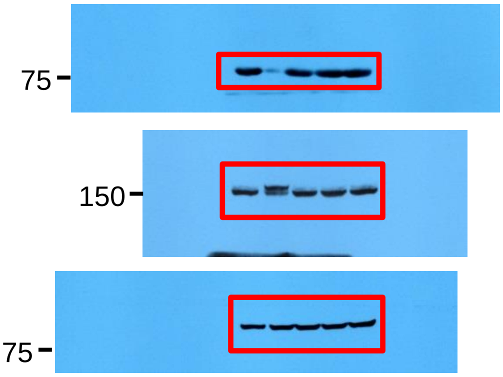

Figure 4c

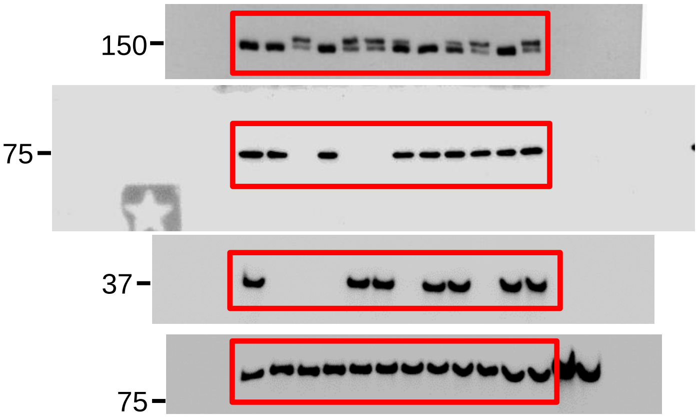

Suppl .Figure 1c

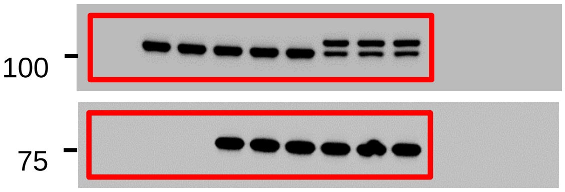

Suppl.Figure 2c

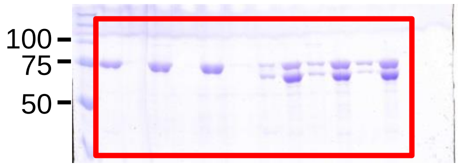

Suppl.Figure 2d

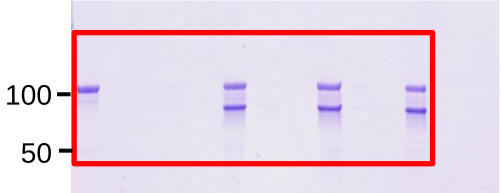

Suppl.Figure 2e

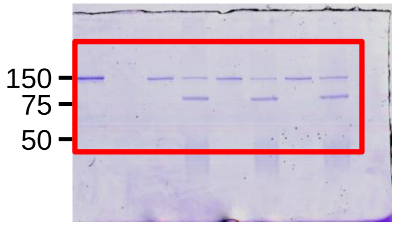

Suppl.Figure 2f

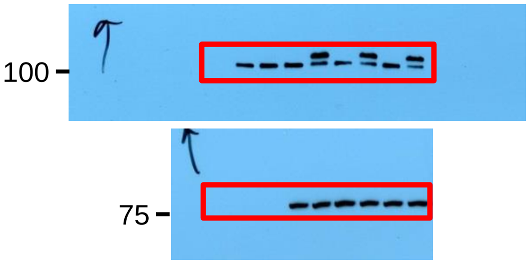

Suppl.Figure 3a

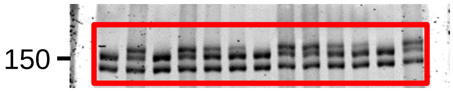

Suppl.Figure 3d

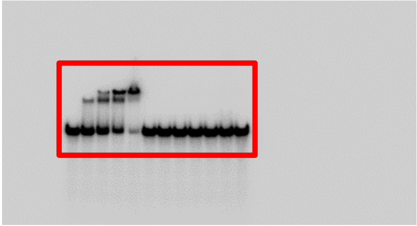

Suppl.Figure 3b

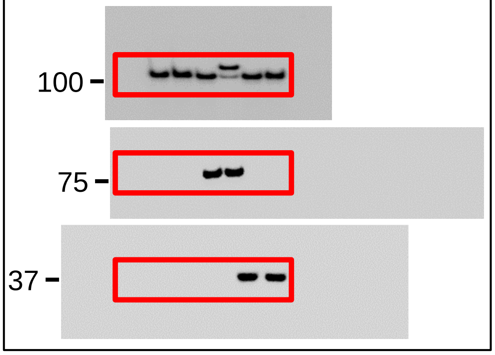

Suppl.Figure 3e

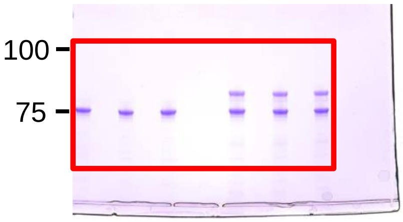

Suppl.Figure 4a

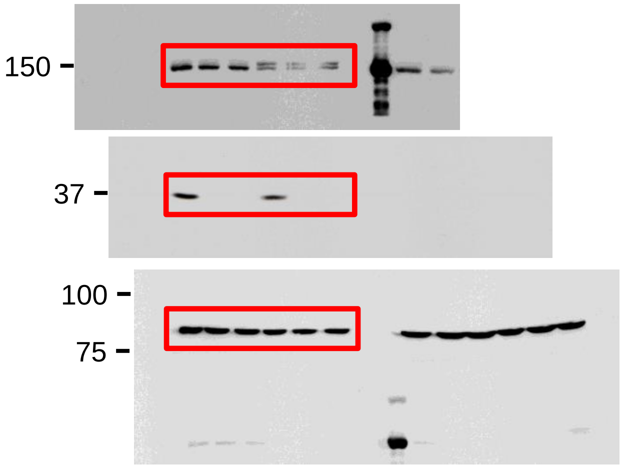

Supplement: Supplementary file 4 — Source Data [file 41467_2019_10408_MOESM4_ESM.zip › Source Data 2 - Uncropped versions of gels and blots presented in the figures.pdf]
